# Editorial: Navigating equality: addressing stigma and discrimination against sexual and gender minorities in access to gender-affirming healthcare

**DOI:** 10.3389/fsoc.2026.1853323

**Published:** 2026-05-04

**Authors:** Evangelos C. Fradelos, Foteini Tzavella

**Affiliations:** 1 Laboratory of Clinical Nursing, Department of Nursing, University of Thessaly, Larissa, Greece; 2 Department of Nursing, University of Peloponnese, Tripoli, Greece

**Keywords:** discrimination, gender minorities, gender-affirming healthcare, healthcare disparities, inclusive practices, LGBTQ+ health, sexual minorities, stigma

In recent years, attention from researchers and clinicians on the topic of health disparities of LGBTQI+ individuals has moved from simply describing differences between groups to trying to understand more deeply and with multiple layers why these disparities exist and persist within today's healthcare systems. This Research Topic aims to support this transition by highlighting the enduring stigma and discrimination experienced by LGBTQI+ individuals within contemporary healthcare systems, notwithstanding the legal recognition achieved in many jurisdictions. The shift from formal equality to the lived experience of care suggests that interpretation must consider multiple dimensions, including normative structures and institutional practices. Minorities stress theory and intersectionality provide a robust theoretical framework for understanding healthcare inequities as the result of multiple and interrelated social forces (Meyer, 2003; Crenshaw, 1989), however full understanding of these experiences requires not only theoretical exploration but also exploration of an individual's phenomenological experience i.e., how they (the individual) experience their own body, their identity, and their relationship to the larger world around them.

The French phenomenological philosopher Maurice Merleau-Ponty considered the body not simply as a biological object, but as the flesh and the fundamental way in which the subject exists and relates in this world (Merleau-Ponty, 1962). Thus, the experience of health is embodied, relational, and deeply meaningful. When a trans or non-binary person enters a healthcare system that operates with strictly binary gender categories, it can become a form of dissonance between lived and imposed identity. This experience is not only organizational or communicative; it is also deeply personal, and it affects how individuals understand and experience themselves. This fact is supported by empirical data where non-binary individuals often experience misgendering and, at the same time, report lower levels of respect, reduced comfort when encountering health professionals (Nowaskie and Menez). Nonetheless, these experiences are not simply isolated incidents of malpractice, but indications of a deeper failure of health systems to recognize the diversity of human experience. Moments like those, Sartre's existential approach offers an additional interpretive tool, as the “gaze of the other” can turn the individual into an object, limiting them to categories that do not reflect their lived reality (Sartre, 2003).

In addition, the concept of dehumanization has become particularly important. In recent research among primary care nurses in Greece resulted that dehumanization toward LGBTQI+ people is not a marginal phenomenon but occurs at moderate levels and is directly related to reduced willingness and comfort in providing care to LGBTQ+ individuals (Bakalis et al., 2025). More specifically, animalistic dehumanization was strongly associated with negative attitudes and reduced professional engagement, which highlights that the attitudes of health professionals are not neutral but directly affect the quality of care provided. The clinical implications of this finding are crucial: when the health professional sees, even unconsciously, the other as a less complete person, less familiar, or less “normal,” then care becomes poorer, more formal, less empathetic, and often less safe.

Among the factors shaping these attitudes toward the personality and psychological characteristics of LGBTQ+ individuals, several are particularly important. In a recent study among nursing students, characteristics such as empathy, emotional stability, and openness to experience shape positive attitudes toward transgender people (Fradelos et al., 2025). This finding has not only academic value, but also valuable educational and clinical implications: the attitudes that professionals convey to care are not shaped exclusively through technical knowledge, but also through deeper personal and emotional processes. Therefore, clinical education cannot remain exclusively biomedical; it needs to include reflection, awareness-raising, and critical processing of prejudices. While, again, in a study among nursing students, it was revealed that significant knowledge deficits exist among nursing students regarding the health needs of LGBTQI+ people (Fradelos et al., 2022). In addition, this finding has direct implications for clinical practice, because insufficient knowledge not only leads to embarrassment or insecurity, but can also translate into poor assessment, inappropriate communication, avoidance of difficult conversations, resulting in poorer quality care. When a professional is unaware of the specific needs, risks, and experiences of a population, they cannot provide truly equitable care, even if their intentions are positive. Furthermore, a mixed study on access to LGBTQIA+ care highlighted an equally critical gap between knowledge and practice, as health professionals demonstrated relevant knowledge, but limited communication skills and low levels of cultural competence (Argyriadis et al., 2023). This is extremely important because in clinical reality, care is not only assessed by what the professional knows but also by how they listen, how they ask, how they respond, and how they make space for the experience of the other.

Cultural competence and communication skills are not “soft” or secondary skills; they are the core of human care (Theodosopoulos et al., 2025). When it comes to sexual and gender minorities, the absence of these skills can lead to silence, concealment of identity, avoidance of services, and delayed help-seeking. Furthermore, as LGBTQ+ people interact with healthcare professionals, phenomena such as medical gaslighting can be observed, where patients' experiences are downplayed or questioned (Nowaskie, Blackwood, and Garcia). Medical gaslighting is associated with forms of epistemic injustice (Fricker, 2007), where the individual is not recognized as a reliable source of knowledge about his or her experience. This is resulting in a lack of trust and alienation from health services. At a structural level, inequalities are not confined to interpersonal relationships; rather, they are reproduced and sustained through institutional practices, policies, and organizational norms within the healthcare system. Experiences of discrimination in insurance agencies and administrative processes demonstrate that inequality is a systemic feature (Nowaskie, Blackwood, Garcia, and Flautero). Intersectionality offers a valuable tool for understanding the complexity of these experiences. Identities are constructed through the intersection of gender, race, socioeconomic status, and sexuality (Zhang et al.), leading to multiple levels of exclusion and inequality (Ikhile et al.). At a macro-social level, the existence of legal recognition does not necessarily imply social acceptance. Cultural and religious narratives continue to influence attitudes and behaviors (Muhire et al.), while stigma operates as a multi-layered phenomenon that is experienced, expected, and internalized (Wuyts et al.). In this context, care cannot be limited to technical skills. The quality of care depends on the professional's ability to be present, to perceive the “here and now” of the patient's experience, and to validate their experience. This recognition is not simply a communicative practice, but a deeply ethical stance. The philosophy of Martin Buber offers a particularly fruitful framework for understanding this relationship. The distinction between “I–Thou” and “I–It” highlights the difference between authentic encounter and objectification (Buber, 1970). In the context of health, the transition to an “I–Thou” relationship is a fundamental prerequisite for building trust and deconstructing stigma.

The Research Topic's articles present various aspects related to the experiences of LGBTQ+ individuals within the healthcare system, ranging from personal to societal to systemic levels of inequity. This collective body of research strongly demonstrates that in order to achieve Health Equity, multi-level and multi-disciplinary approaches must be utilized. Most importantly, overcoming stigma is not enough. Rather than merely address stigma from a political/administrative standpoint, healthcare providers have an ethical and professional obligation to fight against all forms of discrimination (actively) rather than just passively. Healthcare providers must advocate for, bring back the voices of people who have been silenced, increase the visibility of people who have been marginalized, acknowledge their worth, and recognize the value of every person and every identity and every experience; that healthcare is not just an equal or fair privilege only for those individuals who fit the dominant binary/heterosexual norms, but healthcare has to be a universal right and non-negotiable right for all individuals including those" outside the binary or heteronormative norms; as well as all members of the sexual/gender minority communities. Real progress will be seen when health professionals, institutions, and systems stop simply “tolerating” difference and start respecting, including, and caring for it with equality. Only then can care be considered truly humane, fair, and universal.
